# Homemaking away from home: a semiotic cultural psychology perspective

**DOI:** 10.3389/fpsyg.2023.1215654

**Published:** 2023-12-08

**Authors:** Mariann Märtsin

**Affiliations:** School of Governance, Law and Society, Tallinn University, Tallinn, Estonia

**Keywords:** homemaking, migration, culture, meaning-making, geographic and semiotic movement, imagination, semiotic construction, liminal experience

## Abstract

How do migrants create a sense of home in the context of migration? What does it mean for a person to physically move away from one home and psychologically move toward another one somewhere else? How do migrants create a sense of continuity between the home that is no longer there and the home that is not yet here? This theoretical article is an invitation to address these questions from a semiotic cultural psychology perspective. The article emphasizes the importance of both geographical and semiotic movements in understanding the migration process. It shifts the focus away from tangible aspects of migration and toward the imagined and desired aspects of the process of homemaking. The concept of home is explored as a semiotic construction that guides human meaning-making processes, emphasizing its affective value and highlighting the dynamic dialectics of home and non-home. This alternative conceptualization offers new ways of understanding homemaking and being at home, beyond the commonly celebrated ideals of being settled or always being on the move. Finally, the article discusses the dynamic and developmental nature of migration, which can both threaten and open up opportunities for transformation and development, and suggests some general methodological principles that could guide research concerning the interplay between homemaking, migration, and culture.

## 1 Introduction

At the time of writing of this article, the global count of forcibly displaced individuals stands at 108.4 million (UNHCR, 2023). Given the complex landscape of ongoing global socio-political changes, it is increasingly probable that these figures will continue to surge in the foreseeable future. In light of this reality, research exploring the profound significance of home among these displaced populations, as well as others, remains pertinent and compelling.

Since the 1970s, the concept of home has garnered significant interest within various academic fields, first in environmental psychology and later more broadly in social sciences. Over time, home has been studied and conceptualized in diverse ways, reflecting its multifaceted nature. Mallett (2004) writes that the concept of home works as a “repository for complex, inter-related and at times contradictory socio-cultural ideas about people’s relationship with one another, especially family, and with places, spaces and things” (p. 84). In Taylor’s (2015) work, the concept of home is also explored as having multiple dimensions, including its temporal, spatial, material, and relational aspects. Home can be conceptualized as a physical space situated within a specific spatial context. It can be a dwelling, a street, a neighborhood, a city, or a country. It can be a place where one was born or grew up or a place that one has acquired later in life and established as a family home. Home as a physical space can be both tangible and real, as well as intangible and illusory. It can exist as an idealized and imagined space, and is experienced and expressed through embodiment, memory, and imagination. It is a bricolage of varied and multiple bodily, kinesthetic, sensual, and emotional experiences in and of home (Märtsin and Mahmoud, 2012). Home is an important vehicle for building a bridge between past, present, and future (Dahinden, 2012). It provides a means of establishing a sense of continuity throughout a person’s life and also fosters a connection between different generations that have come before and those yet to come. As a relational space, it is a nexus of family life and as such conjures up feelings and experiences of security, comfort, and safety, emerging as a safe haven, a retreat, a place to escape to from the gaze of others, and a place to relax and to refuel (Després, 1991).

However, the concept of home is multifaceted and often contradictory. As feminist writers have pointed out, the connotations of home are not always positive, as this private sphere can also be a site for oppression, violence, and abuse from others (Woodhall-Melnik et al., 2017). Home can conceal traumatic experiences and be associated with fear, danger, and insecurity, particularly for children, young people, and women (Jones, 2000). In some instances, it may become a prison rather than a sanctuary. Home and homeland can be complex and contradictory for refugees too (Womersley, 2020). While they may long for their former home and the familiarity it offers, it can also be a source of fear and violence from which they had to flee. For many refugees, home represents both a cherished memory and a painful reminder of past traumas (Taylor, 2015).

Recent theoretical developments in the area of home have shifted away from attempts to provide a fixed definition or description of home as a static concept. Instead, emphasis is placed on the processual nature of making, unmaking, and remaking home. Home is now viewed as a complex, multifaceted construction that is in a continual state of flux and transformation. Boccagni (2022), for example, suggests the use of the verb “homing,” which emphasizes the processual aspect of homemaking and highlights the active role and efforts of individuals in creating and re-creating their sense of home.

The current article also takes off from this idea that homemaking is a constantly evolving process, which is never truly complete despite individuals’ persistent efforts to make it a reality. My particular focus is on homemaking away from home. How do migrants create a sense of home in the context of migration? What does it mean for a person to physically move away from one home and start to psychologically move toward another one somewhere else? How do migrants create a sense of continuity between the home that is no longer there and the home that is not yet here? To address these questions, I utilize the framework of semiotic cultural psychology. The primary focus of cultural psychology, in its various forms, is to explore the complex systems of human meaning-making, which often become visible in situations where an individual’s everyday conduct is disrupted and new ways of relating to oneself, others, and the world must be constructed to continue working toward future life goals (Valsiner, 2014). Migration presents a distinct and compelling context for cultural psychological investigation, as it underscores the intricate ways in which the movement of human bodies and minds through physical and imaginative spaces shapes and is shaped by cultural meaning systems (Gillespie and Zittoun, 2010). In this theoretical article, I build on these approaches, as well as my own earlier work in the area of migration (Märtsin and Mahmoud, 2012; Märtsin and Samuel, 2023), with the aim of further developing my ideas about home as a hyper-generalized meta-sign and bringing more clearly into focus the ways in which the creation and recreation of meanings of home in the context of migration are guided by migrants’ desires, imaginations, and broader life-goal orientations.

The article is divided into three parts. The first section delves into the essential differentiation between geographic and semiotic movements inherent in the experience of migration, and explores what this distinction implies for our understanding of the homemaking process. In the second part, a cultural psychology perspective on home as a semiotic construction is presented. The third section examines the dynamic and developmental nature of homemaking away from home, highlighting how this experience simultaneously creates ambiguity and threatens our sense of security while also presenting opportunities for transformation and reinvention. The article finishes with some suggestions on how these theoretical ideas can guide the empirical work and methodological choices involved in researching homemaking.

## 2 Homemaking through geographic and semiotic movements

Human beings do not meander through life without direction or purpose; instead, they are constantly striving toward futures they have imagined for themselves (Bühler and Massarik, 1968). We could think about these directions that people choose for their lives as life-goal orientations, formulated not necessarily as concrete plans and goals for the future, but rather as imagined affective fields that we orient ourselves to in our quest to construct meaningful lives for ourselves (Märtsin, 2009). Creating a home away from home can be a cherished goal or aspiration for migrants, as it represents a desire to establish continuity between their past in their homeland and their future in their country of residence, encompasses a sense of belonging to both places, feeling rooted, and connected to the people and culture of both countries. Past experiences become important resources for this movement into the unknown but desired future. The memories of our past homes, together with the affective–relational networks related to these, become essential in imagining the possibility of a new home somewhere else. Even though time moves in an irreversible manner, making the future uncertain and past experiences not directly transferrable to new circumstances, people have the ability to use symbolic means to imagine a different reality to their present circumstances. This creative imaginative process enables individuals to pre-adapt to new situations and challenges in the future. As Valsiner (2014) writes: “What looks as if it entails ‘looking back’ at the given moment is actually ‘looking forward’, thanks to the accessibility of different traces of signs from the past. Within irreversible time one cannot reference ‘what was’ without making it to be in the service of ‘what might come’” (p. 118). Humans thus make meanings ahead of time. We exist as both active participants and objective observers of our context, giving us the unique ability to both distance ourselves from our immediate environment and be influenced by it (Boesch, 1991). As humans, we often envision an ideal version of our lives and use that vision to guide our actions. Moving to another country in search of an imagined, yet not yet realized, safer or improved way of life can be seen as precisely this kind of pre-adaptive action. We construct scenarios and plans based on these life-goal orientations, which motivate us to pursue our goals with purpose and determination, so that we can turn what was imagined into reality (Valsiner, 2014).

Imagination is thus central to human meaning-making. Through imagination, human action is expanded from the *here-and-now* sensations and perceptions to the *elsewhere* and *not-yet-here* sphere of experiences (Levitan, 2019; Glăveanu, 2020; Zittoun, 2020). This movement between present and future, real and possible, here and elsewhere that is made possible by human imagination, creativity, and wonder is central in understanding the experiences of homemaking in the context of migration. The exploration of migration in cultural psychology goes beyond exploring the mere physical relocation from one location to another. It involves a more profound semiotic movement, which takes place on the imaginative level. In fact, this semiotic movement always occurs prior to and in conjunction with the actual geographical relocation. As Gillespie et al. (2012) suggest:

“Geographic movement leads to semantic [i.e., semiotic] movement, both for those who move and for those who receive, and often for many years later. The geographic movement of immigration creates huge semantic reverberations, as all the people affected by the movement reposition themselves in relation to that movement” (pp. 705–706).

Through this kind of semiotic movement, people can engage with their own proximal and distal experiences, but also those of others (Gillespie and Zittoun, 2010). That is, our imagination conjures up thoughts and feelings about how things have been and how they could be for us and for those around us and enables us to turn these into future life-goal orientations. In this way, our dreams about home and migration, among other things, are hooked to those of others. For example, the hopes and desires of a highly educated mother who could not provide the same educational opportunities for her daughters in their home country echo in a young refugee woman’s life-goal orientations as she now lives away from the oppressive regime and seeks to achieve higher education in her new homeland. The act of creating a home away from home thus involves not only the physical markers of home that are brought from the old home to the new place, but also the deeper semiotic and imaginative aspects of what home represents and how it could or should be in the new context. Glăveanu (2020) posits that migration can evoke distinct types of imaginaries, which can either be positive or negative. According to him, the positive imaginaries, which he terms “bright imaginaries,” may involve the allure of change and adventure, the opportunity to leave behind one’s current circumstances and start afresh, or a romanticized vision of a better life for oneself and one’s family (Glăveanu, 2020, p. 72). Conversely, the negative imaginaries of migration, which Glăveanu labels as “dark imaginaries,” may include an individual’s apprehension of losing their life or that of their family members, and their inability to settle down, acquire a livelihood, or attain citizenship. While the bright imaginaries may motivate highly skilled professionals and international students to migrate (see, for instance, Levitan, 2019; Cangià, 2020), dark imaginaries are typical of forced migration due to war, persecution, or climate change (see, for example, Mahmoud, 2014; Womersley, 2020).

In many ways, migration involves a transformation of the self and the reconstruction of one’s sense of identity as individuals create and adopt new perspectives on themselves, others, and the world around them (Märtsin, 2009). Moving away from home and toward an imagined new place creates a journey toward a different way of life and a different way of being, guided by thoughts, emotions, and actions that can turn that imagination into reality. Home, as a semiotic construction, serves as both the point of departure and the imagined endpoint for the migrant, holding experiences of the past and present, as well as hopes and dreams for the future. To fully understand the complexity of the concept of home, it is important to take into account not only the ways in which a person has already constructed their home but also the ongoing and ever-evolving relationship between home and non-home. The exploration of the dynamic interplay between home and non-home offers a nuanced and comprehensive understanding of the complex processes involved in making, unmaking, and remaking home. Migration, as a complex phenomenon, involves a dialectic of leaving and remaining, change and continuity, novelty and everydayness, much like the dialectic from which the semiotic construction of home emerges (Märtsin and Mahmoud, 2012). On the one hand, for an ambitious young man pursuing an academic career abroad, homeland can seem like a stagnant place where his career aspirations and desire to live a cosmopolitan life cannot be achieved. On the other hand, homeland for the same person can also emerge as a place where one can escape from the tensions and pressures of pursuing these same aspirations. Homeland can simultaneously emerge as a cage where one can get stuck and also as a refuge from the pressure of being always on the move—a complex meaning field that guides one’s life and helps one to make sense of everyday experiences (see Märtsin, 2009 for further analysis).

While individuals actively create imaginaries of home and migration, these are always also historically laden and socio-culturally constrained. Culture provides persons with boundaries for their meaning-making, directing them toward the most appropriate and probable approaches to interpreting their experiences and developing aspirations for the future, within a particular socio-cultural context. Out of these bounded possibilities, persons construct their idiosyncratic ways of relating to the world (Salvatore, 2018). In turn, the person’s actualized ways of making meaning become part of the background in relation to which others in the future construct their meanings. Migration, although undertaken by the person, does not occur in a vacuum but is always guided by the socio-cultural meaning potentials available to the individuals. Building on this perspective, one could thus ask how the meaning potentials for conducting one’s life and imagining one’s future change or remain the same as people move across cultural and/or national borders physically and semiotically. The rupture created by the actual or imagined border crossing makes the processes at the border, between known and unknown, between past and future, between what has already been and what is becoming, visible and observable (Valsiner, 2017). It allows examining the process of homemaking as a process at the border, as a process of becoming (Stenner, 2017), and a process of emergence of novelty or maintenance of stability, rather than focusing on meanings of home as already emerged or having been achieved.

## 3 Home as a semiotic construction

Following from the ideas already discussed, it becomes clear that home is not just a physical or geographical location, but a sign or semiotic construction that emerges from the ongoing meaning-making processes including our own and others’ imaginaries related to home and homemaking. In conceptualizing home as a sign and homemaking as part of the meaning-making process, I build on the work of Valsiner (2007) and on my own work on hyper-generalization of signs (Märtsin, 2010). According to this view, signs that hold a significant affective value for individuals and guide the ways they make sense of their relationships with the self, others, and the surrounding world are known as hyper-generalized meta-signs. This category of signs includes values such as freedom or democracy, along with other signs like beauty that evoke a similar powerful affective response (Valsiner, 2007). We know when we are experiencing beauty, even if we are unable to put this experience into words and tell others and ourselves what exactly we are experiencing. Hyper-generalized signs play a crucial role in a person’s intra-psychological semiotic architecture, as they are generalized to the highest level and have the power to regulate the creation, maintenance, or destruction of other signs at lower levels of generalization. This makes them a powerful background against which a person’s meaning-making process unfolds. The strong affective value attached to hyper-generalized signs makes them an integral part of a person’s identity processes and can influence how they perceive and interact with the world around them (Märtsin, 2009).

In my earlier work, I have discussed how a person’s sense of identity can be seen as such a hyper-generalized semiotic construction. In this article, as well as in a recent review chapter (Märtsin and Samuel, 2023), I suggest that home can also be conceptualized as a hyper-generalized meta-sign that operates as an unspoken backdrop to our everyday functioning in the context of migration. My suggestion is that as a hyper-generalized sign, home is an ever-present and powerful regulator of our ongoing meaning-making, including the creation, maintenance, and destruction of imaginaries related to migration. Despite its pervasive influence, we often fail to acknowledge its significance, taking it for granted. Importantly, it only comes to our attention when it is violated, disturbed, or animated—as is often the case in the context of migration. In such situations, meta-sign becomes disrupted and individuals are forced to negotiate new meanings and create new imaginaries.

My proposition is that what emerges in these kinds of situations is the sense of home in relation to a sense of non-home. That is, the meaning of home is inherently tied to its opposite, non-home. It is only through their interdependent and mutually defining antinomies that the meaning of home can be accessed and understood (Märtsin and Mahmoud, 2012). One may not be able to explain what home is, but is able to say when something does not feel like home. Home is animated by the tension between opposing processes of journeying and staying, arrival and departure, feeling secure and insecure, being on the inside and outside, and feelings of being-at-home vs. yearning-for-home. The need for homemaking arises when one feels like a stranger, an outsider in one’s surroundings. Home becomes a place of comfort and peace only in contrast to the discomfort and unease of other places. Through this dynamic interplay, the meaning of home is continually redefined and negotiated, shaped by our experiences and memories of both home and non-home. According to Dovey (1985), to experience the meaning of home is to experience such dialectics: “There is no sense of home unless there is also journeying. […] Without a public realm there is no privacy. And in a sense, without homelessness, we would not be concerned with what home means” (p. 48).

## 4 Making, unmaking, and remaking a home

Migration is a process that is likely to disrupt an individual’s normal flow of functioning and awakens the meaning of home in all its various forms and dimensions. The absence of familiar home-places, the lack of social relationships, and questions regarding one’s identity as it relates to one’s home are suddenly brought to the forefront of consciousness. What was previously taken for granted is now very much present and demands attention. What was taken for granted crumbles in the face of a new reality. With new ways of living yet to be established, uncertainty and ambiguity arise (Märtsin, 2009). Greco and Stenner (2017) describe these situations, where previous structures are no longer applicable but new ones are yet to be implemented, as liminal experiences. They suggest that liminality arises “where an existing form-of-process is suspended (becomes unviable) and a new one is not yet in place” (p. 152). Szakolczai (2015) contends that liminality involves “removing the limit” or existing “at the limit,” resulting in a “genuine Alice-in-Wonderland experience, a situation where almost anything is possible” (p. 18). My suggestion is that the experience of moving toward home away from home can be viewed as precisely this kind of liminal experience, where old ways of being at home and thinking about home are dismantled, while new ways are still in the process of being established.

Liminal experiences resulting from a rupture are characterized by two aspects. First, they generate a significant amount of frustration, tension, and anxiety for individuals involved. When old ways of being disappear, and new ways have not yet emerged, people lose their sense of security and guidance about appropriate behavior. In other words, when the future is uncertain, and the usual signposts that guide behavior no longer function, anxiety prevails, and it becomes challenging to imagine a future that leads out of liminality. Additionally, Szakolczai (2017) suggests that liminality can become permanent, where all action becomes paralyzed, leading to the depletion of resources and ultimately devastation. In the context of migration, these ideas are particularly relevant to the experiences of refugees, who often find themselves stranded indefinitely in refugee camps or processing centers in the countries of first asylum (see Mahmoud, 2014). However, this sense of being trapped and unable to move can also apply to other migrants. Cangià (2020), for instance, discusses the experiences of partners and spouses of professionals who frequently relocate internationally. These “trailing spouses” or “secondary movers” often lose their jobs and career prospects due to their families’ frequent mobility, leaving them stuck in a state of waiting for employment opportunities that can fit within the constraints of their temporary status in their current country of residence.

Nevertheless, liminality is not solely characterized by loss and absence, but is also a state in which new structures of meaning can be created. It is during this state of uncertainty and ambiguity that innovation and creativity can emerge, leading to alternative ways of being and doing (Szakolczai, 2015). Liminal experiences can therefore be referred to as experiences of becoming, for they instigate change and serve as catalysts for transformation. In Cangià’s (2020) study of “trailing spouses,” for instance, some spouses proactively envision new opportunities to remain active and engaged, to provide for their families, and to advance their own careers, such as opening a sports equipment shop or joining a local spousal organization. Hence, the liminality induced by migration should not be automatically viewed as negative and problematic, resulting in unwelcome tensions and changes. Instead, it can be seen as an opportunity for growth and development, leading to novel and more adaptive ways of functioning (Abbey, 2012). Here again, the semiotic moves within the mind precede the actual physical moves out of liminality, preparing the person for life beyond the rupture.

Migration thus creates liminal experiences, where ruptures emerge and transition periods out of these ruptures need to occur for migrants to adapt to their new life circumstances and move toward creating a home away from home. The home will become unmade in this process and will have to be remade, with the possibility that it will never be completely attained again. It is in this sense that migrants will never be at home again. Yet this processual view of homemaking proposed in this article does not posit that all that is real in this process is this constant flow of experiences where everything is always in flux with no solid structures to be established. Instead, my proposal here is that the signs indicating what home is, where it is, with whom it is, and how I am in relation to home are constantly being created in this process, feeding into the process of meaning generalization and hyper-generalization. New signs are constantly created in this process; some already existing signs may be abandoned, while others become reformulated and others further strengthened. These processes of experiencing home away from home and imagining it in relation to one’s changing life-goal orientation in a new place enable the reconstruction of home as a sign until it once again stabilizes in its hyper-generalized form and becomes a powerful background of our everyday functioning.

## 5 Studying the dynamics of homemaking

The conceptualization discussed in this article highlights the processual and dynamic nature of homemaking processes. Migration and homemaking processes are discussed here in terms of their dynamic and developmental nature, both threatening and opening up opportunities for transformation and development. Yet revealing and understanding the semiotic dynamics in the processes of homemaking and migration requires not only new conceptual tools, but also novel methodological approaches that are congruent with the proposed theoretical principles. I conclude this article by briefly suggesting some general methodological principles that could guide the investigation of homemaking processes.

First, this focus on intra-psychological dynamics and understanding how these are guided by the socio-cultural affordances directs the researcher away from a nomothetic approach and toward an idiographic approach in empirical work (see Salvatore and Valsiner, 2010; Toomela and Valsiner, 2010; Valsiner, 2017). It is difficult to envision how the complex semiotic patterns of home and non-home could be revealed through survey or using other methods that aim to investigate average tendencies. The preferred approach is rather one of carefully chosen single case(s) that allow in-depth investigation and lead to novel conceptual insights that can be generalized to other cases. While stemming from the need to engage with processes and understand these in greater depth, this is also well-aligned with the overall purpose in semiotic cultural psychology of developing a general social science perspective that explains how humans as intentional and future-oriented meaning-makers pre-adapt to the world around them (Valsiner, 2014).

Second, the focus on examining and understanding unfolding semiotic processes requires researchers to adopt new dynamic methodologies that are compatible with such a conceptual framework (Valsiner, 2014). In some cases, this has meant turning the gaze toward creative or mobile methods in cultural psychology studies (see Märtsin, 2014; Levitan, 2019; Samuel, 2020). Creative methods can indeed be effective for accessing the unspeakable, for revealing the untold and alternative stories about journeys related to home and non-home (Reavey, 2011). Yet overall, the focus on the process of homemaking means that methods cannot be seen as stand-alone or ready-made tools for collecting data; rather, the construction of methods is deeply intertwined with other aspects of the study, especially basic assumptions about the world, the researcher’s understanding of the phenomena, his or her theoretical concepts, and the data to be collected. The relations between these different aspects, namely, the relations between basic assumptions and phenomena, between theory and method construction, phenomena and method construction, and methods and data, need to be carefully considered and decisions about the best ways of resolving the tensions in these relations need to be reached (Märtsin, 2021). Methods for investigating the processes of homemaking and migration should thus always be constructed depending on the specific research problem at hand. It is in this sense that they are dynamic and developing, always in the process of becoming.

## 6 Conclusion

In this theoretical article, I examined how migrants create a sense of home in the context of migration, proposing a semiotic cultural psychology conceptualization of homemaking processes and discussing the complex interplay between homemaking, migration, and culture. By emphasizing the importance of semiotic movements in addition to geographical, I aimed to shift focus away from the visible and tangible aspects of migration and toward the imagined and desired aspects, as well as people’s efforts to make their life goals or orientations into reality. I conceptualized home as a semiotic construction that guides human meaning-making processes in the context of homemaking and migration, emphasizing its affective value and underscoring how the meanings of home emerge from the dynamic dialectics of home and non-home. I believe that this kind of conceptualization opens up alternative ways of understanding homemaking and being at home, beyond idealized and commonly celebrated meanings of experiencing home as either a desired state of being settled or as a romanticized and privileged condition of always being on the move. I emphasized the importance of understanding the dialectics of change and continuity, mobility and immobility, and conceptualizing migration as a dynamic and developmental process that both threatens and opens up opportunities for development and transformation. I also suggested some general methodological principles that could guide this process-focused approach to investigating homemaking and migration.

While the focus of this paper has been on the intra-psychological processes of moving away from and toward home, I want to conclude with a shift away from the psychological and toward a broader sociological view. As discussed in this article, the utilization of cultural resources in a personal and meaningful manner is vital in the process of transitioning out of liminal experiences that are linked to migration, allowing for the creation of a home away from home. This highlights the important role that culture, with its various rituals, plays in supporting individuals as they geographically or psychologically move across borders and through related transitions (Salvatore and Venuleo, 2017). However, a focus on the cultural scaffolding of migration and homemaking processes can also prompt us to consider intentional integration processes in the new country of residence that are designed for new migrants upon arrival. It raises questions such as: how can these integration processes be designed and accessed so that they are meaningful and useful for migrants; what is the responsibility of the migrant in this process; and where does the responsibility of the receiving community lie in supporting these processes? We know from the vast literature on migration experiences that the process of homemaking, unmaking, and remaking goes beyond finding a physical dwelling or a place of employment in a new place. If so, how can this knowledge be used to scaffold the homemaking processes of new migrants and refugees on a community and societal level? The ways in which a semiotic cultural psychology perspective can make a contribution to providing a deeper understanding of the co-creation and meaning co-construction processes in a community mobilization context is a worthy avenue to explore to further understand the interplay between homemaking, migration, and culture.

## Data availability statement

The original contributions presented in the study are included in the article/supplementary material, further inquiries can be directed to the corresponding author.

## Ethics statement

The studies involving humans were approved by Bath University Department of Education. The studies were conducted in accordance with the local legislation and institutional requirements. The participants provided their written informed consent to participate in this study.

## Author contributions

The author confirms being the sole contributor of this work and has approved it for publication.
